# Association between polymorphism in the *FTO *gene and growth and carcass traits in pig crosses

**DOI:** 10.1186/1297-9686-44-13

**Published:** 2012-04-17

**Authors:** Věra Dvořáková, Heinz Bartenschlager, Antonín Stratil, Pavel Horák, Roman Stupka, Jaroslav Čítek, Michal Šprysl, Anna Hrdlicová, Hermann Geldermann

**Affiliations:** 1 Institute of Animal Physiology and Genetics, Academy of Sciences of the Czech Republic, 277 21 Liběchov, Czech Republic; 2 Department of Animal Husbandry, Czech University of Life Sciences in Prague, 165 21 Prague, Czech Republic; 3 Department of Animal Breeding and Biotechnology, University of Hohenheim, D-70593 Stuttgart, Germany

## Abstract

**Background:**

Independent studies have shown that several single nucleotide polymorphisms (SNP) in the human *FTO *(*fat mass and obesity associated*) gene are associated with obesity. SNP have also been identified in the pig *FTO *gene, among which some are associated with selected fat-deposition traits in F_2 _crosses and commercial populations. In this study, using both commercial pig populations and an experimental Meishan × Pietrain F_2 _population, we have investigated the association between one *FTO *SNP and several growth and carcass traits. Association analyses were performed with the *FTO *polymorphism either alone or in combination with polymorphisms in flanking loci.

**Methods:**

SNP (FM244720:g.400C>G) in exon 3 of porcine *FTO *was genotyped by PCR-RFLP and tested for associations with some growth, carcass and fat-related traits. Proportions of genetic variance of four pig chromosome 6 genes (*FTO*, *RYR1*, *LIPE *and *TGFB1*) on selected traits were evaluated using single- and multi-locus models.

**Results:**

Linkage analysis placed *FTO *on the p arm of pig chromosome 6, approximately 22 cM from *RYR1*. In the commercial populations, allele *C *of the *FTO *SNP was significantly associated with back fat depth and allele *G *with muscling traits. In the Meishan × Pietrain F_2 _pigs, heterozygotes with allele *C *from the Pietrain sows and allele *G *from the Meishan boar were more significantly associated with fat-related traits compared to homozygotes with allele *G *from the Pietrain and allele *G *from the Meishan breed. In single- and multi-locus models, genes *RYR1*, *TGFB1 *and *FTO *showed high associations. The contribution in genetic variance from the polymorphism in the *FTO *gene was highest for back fat depth, meat area on the *musculus longissimus lumborum et thoracis *tissues and metabolite glucose-6-phosphate dehydrogenase.

**Conclusions:**

Our results show that in pig, *FTO *influences back fat depth in the commercial populations, while in the Meishan × Pietrain F_2 _pigs with a *CG *genotype, heterosis occurs for several fat-related traits.

## Background

The *fat mass and obesity associated *(*FTO*) gene encodes a protein of unknown function in an unknown pathway. The abbreviation *FTO *originates from a study in mice with a fused toes (*Ft*) phenotype and other abnormalities resulting from a 1.6 Mb deleted region on mouse chromosome 8 that includes this gene. It has been suggested that mouse *Fto *could be involved in programmed cell death, limb development, craniofacial development and the control of left-right asymmetry [1-3]. *Fto *mRNA is expressed in all murine tissues examined, with the highest signal detected in the brain and more specifically in the hypothalamus, which plays a key role in the control of energy balance [4].

The human *FTO *gene is more than 400 kb long and is located on human chromosome 16q12.2 http://www.ensembl.org/Homo_sapiens. Sequence analysis has shown that the encoded protein, FTO, shares amino-acid motifs with the Fe(II)- and 2-oxoglutarate-dependent oxygenases, which are involved in various processes, including DNA repair, fatty acid metabolism and posttranslational modifications [4,5]. Wu et al. [6] have reported that FTO is a transcriptional co-activator, which facilitates transcription from unmethylated and methylation-inhibited gene promoters and enhances C/EBPs binding to DNA, and that it may play a role in the regulation of adiposity.

Studies on obesity-associated genes in humans [7-9] have identified several single nucleotide polymorphisms (SNP) in *FTO *intron 1 that are associated with obesity. Additional studies have confirmed these results e.g. [10,11] but not for all ethnic groups (see reviews [12,13]).

The porcine *FTO *gene has been mapped to the p arm of chromosome 6 or SSC6 (SSC for *Sus scrofa*) by radiation hybrid mapping and linkage analysis [14-16]. Whilst the porcine *FTO *gene is annotated in the Sscrofa9 draft genome assembly (see: http://www.ensembl.org/Sus_scrofa/), unfortunately this sequence is missing from the assembly Sscrofa10.2 on which the pig genome sequence paper will be based ([17]; A. L. Archibald, personal communication; http://www.ncbi.nlm.nih.gov/gene/100127165). Several polymorphisms have been detected in porcine *FTO *[15,18,19] and used in association analyses with selected growth and fat-related traits. Fan et al. [18] have reported the existence of two SNP in this gene, one in intron 1 and one in exon 3 (a synonymous mutation) that are significantly associated (*P *< 0.01) with average daily gain on test and total lipid percentage in muscle, respectively, in a Berkshire × Yorkshire F_2 _population. Fontanesi et al. [15] have identified another polymorphism in intron 4 associated with intermuscular fat deposition in the Duroc breed and with feed conversion rate in Italian Large White pigs. These results have been confirmed in subsequent analyses on Italian Duroc (*P *< 0.01) and commercial pig populations (*P *< 0.05; [16]). Finally, a significant association (*P *< 0.05) has been found between an SNP located in the 5' flanking region of *FTO *and intramuscular fat content in the Jinhua × Pietrain F_2 _reference population [19].

Our study was primarily aimed at investigating the association between an SNP in porcine *FTO *exon 3 and growth, carcass and fat-related traits in Meishan × Pietrain F_2 _and commercial pig populations. We also estimated the proportions of variance of four linked genes on SSC6 (*FTO*, *RYR1*, *LIPE *and *TGFB1*) on selected traits in the Meishan × Pietrain F_2 _population using single- and multi-locus models.

In pig, the *RYR1 *(ryanodine receptor 1) gene has a role in stress resistance and also affects carcass and meat quality traits. Homozygotes carrying the *1843 T *allele (stress sensitive) have heavier, shorter and leaner carcasses than homozygotes carrying the *1843 C *allele (stress resistant). Allele *1843 T *has a near additive effect on lean content, killing-out percentage and carcass length [20]. The protein encoded by the *LIPE *(hormone-sensitive lipase) gene catalyzes the intracellular hydrolysis of triacylglycerols and cholesteryl esters, and is involved in regulating body fat, steroidogenesis and insulin secretion. Population and genetic studies in humans have suggested that genetic variability in *LIPE *could be involved in lipid metabolism and risk for obesity and type 2 diabetes [21]. The *TGFB1 *gene encodes a multifunctional peptide that controls proliferation, differentiation and other functions in many cell types. Rosmond et al. [22] and Long et al. [23] have reported associations between an SNP in *TGFB1 *and variation in obesity phenotype, suggesting a possible effect of the gene on obesity.

## Methods

### Animals and performance traits for association analysis

DNA sampled from unrelated animals of eight pig breeds (Czech Large White, 28; Czech Landrace, 38; Czech Meat Pig, 15; Pietrain, 23; Black Pied Prestice, 17; Hampshire, 9; Duroc, 30; Meishan, 22) and wild boar (10) was used to estimate the frequencies of *FTO *alleles.

Linkage mapping was performed on the Hohenheim Meishan × Pietrain three-generation pedigree [24] using the CRI-MAP software, version 2.4 [25].

An association analysis between *FTO *genotypes and production traits was performed on two populations: (*i*) F_2 _animals from the Hohenheim Meishan × Pietrain pedigree (described in detail by [24], and (*ii*) commercial pigs, mostly originating from crosses (Table 1).

**Table 1:** Pig crossbreds used for association analysis

Group	Pig crosses	*n*
1	PNx(CZLxCZLW)	156
2	CZLW	22
3	CZLWxCZL	23
4	(CZLWxPN)x(CZLxCZLW)	36
5	PICx(CZLxCZLW)	36
6	FHxPIC	60
7	(DxCZLW)x (CZLxCZLW)	72
8	(PNxCZL)x(CZLxCZLW)	64

*PN *Pietrain; *CZL *Czech Landrace; *CZLW *Czech Large White;

*PIC *Pig Improvement Company; *FH *French hybrids; *D *Duroc

Briefly, the Meishan × Pietrain pedigree was obtained from one Meishan boar and eight Pietrain sows as founder animals and consisted of 22 F_1 _animals (three boars and 19 sows) and 316 F_2 _animals raised in uniform environmental conditions at an experimental station at the University of Hohenheim (Germany). Pigs were slaughtered at 210 days of age (SD ± 7.3 days) and had an average live weight of 96.1 kg. One hundred and forty-eight traits were recorded including eight growth and fattening traits, 11 fat deposition traits, eight muscling traits, 14 meat quality and stress resistance traits, and many other specific traits (muscle fibres, metabolites and protein content of the muscle, enzyme activities, fat cell number and volume, etc.).

The 469 commercial pigs were maintained in air-conditioned stables of the Test Station in Ploskov of the Czech University of Life Sciences in Prague under standard conditions. At the start of the test, pigs had a live weight of 25-30 kg and were slaughtered at a target weight of 113 kg (SD ± 10.5). They were fed a commercial diet (wheat, barley, soybean meal and a premix of supplements of essential elements) *ad libitum *and dosed feed. The following performance traits were recorded: average back fat depth (calculated from back fat depth 1 i.e. above the first thoracic vertebra, back fat depth 2 i.e. above the last thoracic vertebra, back fat depth 3 i.e. above the first lumbar vertebra); intramuscular fat in *m. longissimus lumborum et thoracis *(*m.l.l.t*.) and fat content of belly 2 (for information on belly 2, see [26]; the method used a gravimetric determination according to the Czech norm ISO 1443); fat and muscle depth and lean meat (measured with Fat-O-Meter); feed conversion. The average daily gain during the test was calculated as the ratio between live weight gained from the beginning to the end of the test and corresponding days.

Due to practical and organizational reasons, individual performances were not recorded for all the animals, which explain the difference in numbers of pigs analysed for the different traits.

All pigs were slaughtered according to protocols for certified European (Germany) or national (Czech Republic) slaughterhouses under the control of an independent veterinarian.

### PCR amplification and genotyping of *FTO*, *RYR1*, *LIPE *and *TGFB1*

To identify polymorphisms in the porcine *FTO *gene, polymerase chain reaction (PCR) primers were designed based on the porcine cDNA *FTO *sequence corresponding to exon 3 of human *FTO *[Ensembl:ENSG00000140718]. The SNP reported in *FTO *intron 4 by [15] was also genotyped in commercial pig breeds and crosses. Using single- and multi-locus models, the proportions of variance of three linked genes (*RYR1*, *LIPE *and *TGFB1*) were estimated in the Meishan × Pietrain pedigree. Additional file 1 contains information on PCR-RFLP conditions for *FTO, RYR1*, *LIPE *and *TGFB1 *[see Additional file 1]. *FTO *PCR products obtained from wild boar, Pietrain and Meishan DNA were sequenced (ABI PRISM 3130 Sequencer; Applied Biosystem, Foster City, CA, USA) and the sequences were deposited in the public database [EMBL: FM244719 - FM244721].

### Association analysis

Associations between *FTO *SNP FM244720:g.400C>G and quantitative traits were analysed with SAS^® ^(SAS^® ^Inst. Inc., Cary, NC) using SAS^® ^8.2 Version and the results of the Type III sum of squares calculation for the Meishan × Pietrain pedigree, and the GLM procedure (Type IV) of SAS 9.1 for the commercial populations.

(*i*) For the statistical evaluation of the Meishan × Pietrain F_2 _family, the following model A was used:

(A) $${\text{Y}}_{\text{ijklm}}=\;\mu \;+\text{ FT}{\text{O}}_{\text{i}}+\text{ mont}{\text{h}}_{\text{j}}+\text{ se}{\text{x}}_{\text{k}}+\text{ littern}{\text{o}}_{\text{l}}+\text{ b}\left(\text{s}\text{\_}\text{ag}{\text{e}}_{\text{ijklm}}-\text{ S}\text{\_}\text{age}\right)\;+\;{\text{e}}_{\text{ijklm}}$$

where Y_ijklm _= value of a trait for animal m; μ = estimated mean value of a trait; FTO_i _= the effect of genotype class for *FTO *(*C*m*C*m, *C*m*G*m, *C*m*G*p, *G*m*G*p, *G*p*G*p) with *C*m*C*m being: homozygotes with both alleles *C *of grandmaternal origin; *C*m*G*m: heterozygotes with alleles *CG*, both of grandmaternal origin; *C*m*G*p: heterozygotes with alleles *CG *inherited from both grandparents; *G*m*G*p: homozygotes with alleles *GG *inherited from both grandparents; *G*p*G*p: homozygotes with both alleles *G *of grandpaternal origin (the genotype classes for *FTO *were created using the procedure Chrompic from the CRI-MAP package [25]. The maternal and paternal phase information for all animals is marked with signs for grandmaternal or grandpaternal origin for all loci included in the map calculation); month_j _= the effect of two-month class created from slaughter date (j = 1-6); sex_k _(k = 1, 2); litterno_l _= litter number (l = 1, 2); b = linear regression value; s_age_ijklm _= age at slaughter of animal m; S_age = estimated average of the age at slaughter; e_ijklm _= random residual.

Models including different genes (*FTO*, as well as *RYR1*, *LIPE *and *TGFB1 *- for more information, see [27]) with their genotype classes in the initial model (starting model including all non genetic independent variables) were tested stepwise to find the highest proportion of variance reduction (VR). In cases for which inclusion of another locus in the model did not result in any increase of VR (%), only a single-locus model was retained (e.g. for meat area on *m.l.l.t*.). Genes that showed no significant association remained in a combined model if their contribution to the total VR reached about 2%.

(*ii*) For analyses on commercial pigs, the model included the *FTO *genotype with/or without the *RYR1 CT *genotype, a crossbred combination, gender and type of diet as fixed factors, and average of age at slaughter as a regression coefficient. The statistical model B was:

(B) $${\text{Y}}_{\text{ijklmn}}=\mu \;+{\text{ FTO}}_{\text{i}}+{\text{ RYR}}_{\text{j}}*\;+{\text{ cross}}_{\text{k}}**\;+{\text{ sex}}_{\text{l}}+{\text{ diet}}_{\text{m}}+\text{ b(s}\_{\text{age}}_{\text{ijklmn}}-\text{ S}\_\text{age) }+{\text{ e}}_{\text{ijklmn}}$$

where: Y_ijklmn _= value of a trait for animal n; μ = estimated mean value of a trait; FTO_i _= the effect of *FTO *genotype (i = 1, 2, 3); RYR_j _= the effect of *RYR1 *genotype (j = 1, 2); cross_k _= the effect of crossbred combination (k = 1, 2, 3, 4, 5, 6, 7, 8); sex_l _(l = 1, 2); diet_m _= the effect of diet (m = 1, 2); b = linear regression value; s_age_ijklmn _= age at slaughter of animal n; S_age = estimated average of age at slaughter; e_ijklmn _= random residual. * means that the association analysis of animals with genotype *CC *at gene *RYR1 *did not include the effect of gene *RYR1 *and ** means that the separate association analysis of crossbred combination PNx(CZLxCZLW) did not include the effects of other crossbred combinations.

The differences between genotype classes were assessed by *t*-test.

## Results and discussion

### Polymorphism, allele frequencies and linkage analysis of the *FTO *gene

Sequence analyses identified two SNP, FM244721:g.307C>T and FM244720:g400C>G that were detected by PCR - restriction fragment length polymorphism (RFLP) using conditions presented in Additional file 1 [see Additional file 1]. Both SNP are synonymous mutations localized in exon 3. SNP g.400C>G is identical to SNP c.594C>G as reported by Fan et al. [18].

Pigs of different breeds were analysed for SNP FM244720:g.400C>G (as well as for intron 4 SNP AM931150:g.276T*>*G [15]) and allele frequencies are presented in Table 2.

**Table 2:** Allele frequencies of *FTO *SNP FM244720:g.400C>G in eight pig breeds and the wild boar^1^

Breed	*n*	*C*	*G*
Czech Large White	28	0.45	0.55
Czech Landrace	38	0.66	0.34
Czech Meat Pig	15	0.80	0.20
Pietrain	23	0.65	0.35
Black Pied Prestice	17	0.47	0.53
Hampshire	9	0.94	0.06
Duroc	30	0.43	0.57
Meishan	22	0.02	0.98
Wild boar	10	0.10	0.90

^1 ^Included are pigs that were genotyped for both SNP FM244720:g.400C>G and SNP AM931150:g.276T>G [15]; both SNP were in complete linkage disequilibrium (allele *g.400 C *with *g.276 T*, and *g.400 G *with *g.276 G*), with the exception of one Meishan pig, which was heterozygous *g.400 CG *and homozygous *g.276 GG *(repeatedly tested)

Linkage analysis in the Hohenheim Meishan × Pietrain family placed the *FTO *gene into the previously constructed chromosome 6 linkage map [28] and the most probable order (Kosambi cM; sex averaged) was: *S0035 *- 24.5 - *SW1329 *- 33.7 - *SW1057 *- 16.4 - ***FTO ***- 6.4 - *S0087 *- 15.7 - *RYR1 *- 1.9 - *LIPE *- 0.9 - *TGFB1 *- 1.2 - *A1BG *- 1.4 - *EAH *- 3.4 - *SKI *- 15.8 - *FABP3 *- 3.6 - *ID3 *- 14.2 - *S0146 *- 9.8 - *S0003 *- 16.3 - *SW824 *- 50.1 - *P3 *- 20.0 - *EAO*.

This assignment agrees with earlier mapping results [14-16,19] and the *FTO *locus is most probably situated in the proximal part of the p arm since microsatellites *SW1057 *and *S0087 *have been mapped in this region [29,30]. In both the Sscrofa9 and Sscrofa10.2 draft assemblies of the pig genome, the order is *FTO *- *RYR1 *- *TGFB1 *- *LIPE *- *A1BG*, which is consistent with that of the human orthologs on chromosome 19. In contrast, the order of *TGFB1 *and *LIPE *is reversed in our linkage map. This inconsistency could be due to the short distance between the two genes (~50 kb) and the difficulty of discriminating between alternative orders by linkage analysis with limited numbers of informative meioses. However, the order of tightly linked loci has no influence on the results of genotype effects in single- and multi-locus analyses.

### Association analysis

#### Meishan × Pietrain F_2 _family

The *FTO *g.400C>G genotypes were *GG *in the grandsire (Meishan) and *CC*, *CG *and *GG *in the granddams (Pietrain breed), respectively. The genotype of the three F_1 _boars was *CG *with alleles *C *and *G *inherited respectively from the Pietrain and Meishan grandparents. Since the genes *FTO *and *RYR1 *are linked (separated by ~22 cM) and since *RYR1 *is known to affect several carcass traits, it had to be considered as well. The grandsire's genotype at *RYR1 *was *CC *and all eight granddams were *TT *homozygotes [28].

The genotypes at *FTO *of the grandparents and parents of the F_2 _animals combined with the *FTO *flanking phase information made it possible to identify the grandparental origin of alleles in the F_2 _animals. The results of the association analysis for the genotype classes according to the grandparental alleles' origin are presented in Table 3. The genotype at *RYR1 *was not included as a co-factor in these analyses. If it was included as another factor in the model, the differences between estimates in the one-locus and two-locus models remained (data not shown). We consider that the recombination frequency (0.17) observed between *FTO *and *RYR1 *in this population makes it possible to detect specific associations between *FTO *and the traits analysed, mostly or totally independent from *RYR1*.

**Table 3:** Association analysis between *FTO *SNP FM244720:g.400C>G and selected traits in Meishan × Pietrain F_2 _pigs

Trait	*C*m*C*m^1 ^± SE (*n *= 32)	*C*m*G*m ± SE (*n *= 46)	*C*m*G*p ± SE (*n *= 105)	*G*m*G*p ± SE (*n *= 62)	*G*p*G*p ± SE (*n *= 71)	*P*
Average daily gain (110-210 days of age; g/day)	579 ± 24.75	605 ± 19.87	598 ± 12.98	583 ± 17.38	579 ± 15.77	0.31
Abdominal fat weight (kg)	0.67 ± 0.07^a^	0.72 ± 0.05^ac^	0.97 ± 0.04^b^	0.85 ± 0.05^cd^	0.86 ± 0.04^d^	<0.0001
Back fat depth on *m.l.l.t*. at 13th-14th rib (mm)	18.94 ± 1.09^a^	19.0 ± 0.87^a^	23.58 ± 0.57^b^	21.17 ± 0.76^ac^	22.74 ± 0.69^bc^	<0.0001
Average back fat depth (mm)	25.69 ± 1.08^ac^	25.09 ± 0.87^a^	29.91 ± 0.57^b^	26.93 ± 0.76^ac^	27.98 ± 0.69^c^	<0.0001
Shoulder fat depth (mm)	33.49 ± 1.29^a^	34.27 ± 1.03^a^	38.64 ± 0.68^b^	35.99 ± 0.90^ac^	37.40 ± 0.82^bc^	0.0004
Fat depth at 10th rib (mm)	22.27 ± 1.03^ac^	20.67 ± 0.82^a^	25.30 ± 0.54^b^	22.23 ± 0.72^ac^	23.37 ± 0.65^c^	<0.0001
Loin fat depth (mm)	21.32 ± 1.27^ac^	20.34 ± 1.02^a^	25.80 ± 0.66^b^	22.57 ± 0.89^ac^	23.17 ± 0.81^c^	<0.0001
Fat area on *m.l.l.t*. at 13th-14th rib (cm^2^)	18.43 ± 0.98^a^	19.28 ± 0.78^ac^	22.30 ± 0.51^b^	20.01 ± 0.69^ac^	21.18 ± 0.62^bc^	0.0004
Lean cuts (%)	47.57 ± 0.67^a^	48.10 ± 0.57^a^	44.68 ± 0.35^b^	45.35 ± 0.47^b^	44.20 ± 0.43^b^	<0.0001
Feed consumption (110-210 days of age; kg)	209 ± 7.06^a^	214 ± 5.67^a^	230 ± 3.70^b^	218 ± 4.96^a^	223 ± 4.50^ab^	0.006
Feed conversion (kg/kg)	3.73 ± 0.16^ab^	3.60 ± 0.13^a^	3.94 ± 0.09^b^	3.82 ± 0.11^ab^	4.05 ± 0.10^b^	0.008

Model (A) was used; for each genotype the LS mean ± SE are given

^1 ^m: allele of grandmaternal origin (Pietrain); p: allele of grandpaternal origin (Meishan)

^a,b,c^: values with different letters in the rows differ significantly (*P *< 0.05)

*m.l.l.t*.: *musculus longissimus lumborum et thoracis*

*P*: most significant probability of *t *test between two genotype classes

In Table 3 attention should be paid to the *FTO *genotype classes *C*m*G*p and *G*m*G*p. In both cases, allele *G*p is of grandpaternal origin, and the two classes differ by *C*m and *G*m alleles originating from granddams (Pietrain). Table 3 shows that many fat-related traits (abdominal fat weight, back fat depth on *m.l.l.t*. average back fat depth, shoulder fat depth, fat depth at 10th rib, loin fat depth and fat area on *m.l.l.t*.) as well as feed consumption in the two classes differ significantly, with higher values for genotype *C*m*G*p (i.e. with the *C *allele coming from Pietrain). The values for fat-related traits in this class are also significantly higher than those in classes *C*m*C*m and *C*m*G*m (i.e. with both alleles of grandmaternal origin).

The high positive effects in the *C*m*G*p class most probably represent a heterosis effect, often overdominant in comparison with other classes. However, it may not necessarily be the effect of the *FTO *genotype itself, since extensive linkage disequilibrium in this population means that in the F_2 _generation extensive haplotypes are inherited intact from the Pietrain and Meishan grandparents in the observed chromosome region, and thus other linked genes may be responsible for the heterosis effect on the fat traits. The heterosis effect was apparent when allele *C*m (or an allele in linkage disequilibrium with this) was present. However, the chromosome segments from both breeds led to heterosis, but if both *FTO *alleles (i.e. chromosome segments) come from the Pietrain breed (genotype *C*m*G*m), no heterosis occurred. This effect may be the result of the genetic background of the founders and consequently F_2 _pigs, and may be different in other populations.

Proportions of trait variance associated with the *FTO *gene and three distally located genes (*RYR1*, *LIPE *and *TGFB1*), in single- and multi-locus models for several traits are summarised in Additional file 2 [see Additional file 2]. Three genes (*RYR1*, *TGFB1 *and *FTO*) show strong associations in single- and multi-locus models. The covariance components (interaction effects) of *RYR1 *and *TGFB1 *are often very low and indicate that combining both loci does not increase the declared variance proportion. These genes are also similarly associated with some traits, e.g. fat cuts, lean cuts and meat quality traits. The most significant variance proportion is observed for *FTO *in combination with *RYR1*, *LIPE *and *TGFB1 *in the multi-locus model for back fat depth, whereas for meat area on *m.l.l.t*., and the metabolic trait G6P in fat tissue, it is highest in the single- and multi-locus models, respectively. *TGFB1 *shows the most significant gene effects on weight of ham relative to half carcass weight in both model types.

#### Commercial populations

Commercial crossbred pigs were evaluated in two analyses: (i) with the crossbred combination PNx(CZLxCZLW) alone and (ii) with the combined populations (Table 1). All animals were genotyped for SNP g.400C>G and SNP g.276T>G (see Additional file 1). There was complete linkage disequilibrium between the two loci, giving two haplotypes: *g.400 C*-*g.276 T *and *g.400 G*-*g.276 G*. We used SNP g.400C>G for the analysis.

#### Crossbred combination PNx(CZLxCZLW)

All three *FTO *SNP g.400C>G genotypes were observed. Using model (B) with the genotype at *RYR1 *included, *FTO *SNP g.400C>G was significantly associated with average back fat depth (*P *< 0.0001), fat in the belly 2 (*P *= 0.002), muscle depth (*P *= 0.02) and average daily gain (*P *= 0.02). The highest values for fat traits were observed in animals with genotype *CC *and for muscle depth in those with genotype *GG *(Table 4). If only the genotype *CC *at *RYR1 *was used in the *FTO *association analysis, the results were very similar (data not shown). For intramuscular fat in ham, the number of pigs analysed was rather low (Table 4), so the results are inconclusive.

**Table 4:** Association analysis between *FTO *SNP FM244720:g.400C>G and carcass traits in crossbred pigs

Trait	PNx(CZLxCZLW)^1^				Joint population^1^			
	
	*CC *± SE(*n*)	*CG *± SE(*n*)	*GG *± SE(*n*)	*P*	*CC *± SE(*n*)	*CG *± SE(*n*)	*GG *± SE(*n*)	*P*
Average daily gain (g/day)	801 ± 16.53^a^ (64)	825 ± 13.59 (71)	864 ± 21.55^b^ (20)	**0.02**	873 ± 12.73 (148)	869 ± 10.92^a^ (218)	895 ± 12.35^b^ (99)	**0.02**
Fat in the belly 2 (%)	39.07 ± 2.68^A^ (14)	30.73 ± 1.72^B^ (25)	34.74 ± 2.51 (9)	**0.002**	33.25 ± 1.49^a^ (50)	30.81 ± 1.13^b^ (99)	33.46 ± 1.25^a^ (46)	**0.03**
Intramuscular fat in the *m.l.l.t*. (%)	1.58 ± 0.16 (14)	1.47 ± 0.11 (27)	1.57 ± 0.16 (7)	0.46	1.67 ± 0.13 (38)	1.68 ± 0.10^a^ (78)	1.93 ± 0.12^b^ (28)	**0.04**
Intramuscular fat in the ham (%)	2.18 ± 0.52^a^ (6)	3.52 ± 0.30^b^ (23)	3.29 ± 0.42 (8)	**0.02**	3.01 ± 0.39 (24)	3.42 ± 0.27 (62)	3.42 ± 0.31 (30)	0.29
Average back fat depth (mm)	27.96 ± 0.98^α^ (30)	27.01 ± 0.69^α^ (45)	21.38 ± 1.01^β^ (15)	**< 0.0001**	26.06 ± 0.65^a^ (115)	25.94 ± 0.54^a^ (188)	24.68 ± 0.62^b^ (93)	**0.02**
Fat depth (FOM; mm)	15.76 ± 0.59 (64)	15.30 ± 0.48 (71)	14.97 ± 0.76 (20)	0.41	17.78 ± 0.59 (107)	17.85 ± 0.50^a^ (149)	16.76 ± 0.59^b^ (67)	**0.05**
Lean cuts (FOM; %)	56.80 ± 0.46 (64)	57.32 ± 0.38 (71)	58.03 ± 0.60 (19)	0.11	54.52 ± 0.45^a^ (106)	54.72 ± 0.38^a^ (147)	55.72 ± 0.45^b^ (67)	**0.013**
Muscle depth (FOM; mm)	64.44 ± 1.15^a^ (64)	66.92 ± 0.95^b^ (71)	69.05 ± 1.50^b^ (20)	**0.02**	58.67 ± 1.00^A^ (108)	59.80 ± 0.84 (149)	61.51 ± 1.00^B^ (67)	**0.007**
Feed conversion (kg/kg)	2.89 ± 0.06 (64)	2.91 ± 0.05 (71)	2.80 ± 0.08 (20)	0.22	2.82 ± 0.04^a^ (133)	2.89 ± 0.04^b,A^ (180)	2.78 ± 0.04^B^ (70)	**0.003**

Model (B) *RYR1 *included was used; for each genotype the LS mean ± SE are given;

^1 ^see Table 1; ^a,b; A,B; α,β ^- values with different letters in the rows per animal group differ significantly (*P *< 0.05; *P *< 0.01; *P *< 0.001, respectively); *m.l.l.t*.: *m. longissimus lumborum et thoracis*; *P *- most significant probability of *t *test between two genotype classes

#### The joint population

All three *FTO *SNP g.400C>G genotypes were also observed in the combined commercial pig populations. Using model (B), significant associations were found between the *FTO *SNP and average daily gain (*P *= 0.02), average back fat depth (*P *= 0.02), muscling traits (lean cuts, *P *= 0.013; muscle depth, *P *= 0.007) and feed conversion (*P *= 0.003). In this case too, higher values for back fat depth and fat depth were observed in animals with genotype *CC *and *CG*. For the muscling traits, genotype *GG *was the most favourable (Table 4). Significant associations were also found for fat in the belly 2 and intramuscular fat in the *m.l.l.t*. Values for average daily gain and feed conversion were higher in pigs with genotype *GG *(Table 4). If only the *CC *genotype at *RYR1 *was used in the *FTO *association analysis, the results were similar (data not shown).

Clearly, the quantitative traits studied here are under polygenic control. Tests for associations with a single gene can reveal both the effects on genetic variance of the genotypes at the gene of interest and at the genes in linkage disequilibrium with the gene of interest, only if the animals in the population analysed have similar genetic backgrounds (e.g. pure bred populations) and are subject to standardized environmental influences. These requirements are not always met, mainly for economic reasons. In our experiment, we used the commercial crossbred population PNx(CZLxCZLW) separately, in which the heterogeneity in genetic background is not so high as in the population composed of several crossbreds. However, in both this crossbred population and in the joint population composed of several crossbreds, the *FTO *SNP g.400C>G genotypes *CC *and *CG *were significantly associated with high values for average back fat depth. A similar situation was observed for associations between genotype *GG *and high values for muscle depth.

Effect of genetic background can be observed on F_2 _pigs from the Meishan × Pietrain family, in which the highest effects on fat-related traits, apparently overdominance, are detected in heterozygotes with allele *C *inherited from the Pietrain and allele *G *inherited from the Meishan breed.

Since extended Pietrain and Meishan haplotype blocks are inherited intact in the F_2 _pigs, it was important to include in the models the genotypes of linked genes, which are known to have effects on the traits of interest, especially *RYR1*. We examined the proportion of genetic variance explained by *FTO *and the linked loci. In this case too, highly significant associations between *FTO *polymorphisms and back fat depth, and also meat area on *m.l.l.t*. and G6P were observed. It is hypothesized that other loci on different chromosomes may also be significantly involved in the determination of each trait, as additional proportions of genetic variance (examples are given in [27]).

Previous association analyses between pig *FTO *genotypes and fatness and growth rate traits have been performed with SNP from various regions of the gene [15,16,18,19]. Although different populations were used and different traits were recorded, in all cases, genotypes at some of the SNP were associated mostly with fatness traits. In addition, this study revealed a significant effect of SNP g.400C>G (which is the same as SNP c.594C>G of Fan et al. [18]) on average back fat depth, with genotypes *CC *and *CG *having higher effects.

Associations between the intronic SNP (g.276T>G) and back fat thickness have been reported for Italian Duroc and commercial pig populations [16] and agree with our results on commercial populations. Allele *g.400 C *(and *g.276 T*) is associated with higher values for fat deposition, while allele *g.400 G *(and *g.276 G*) with higher values for meat traits. However, Fan et al. [18] and Du et al. [31] did not find any association between polymorphisms in exon 3 and the 5' untranslated region of porcine *FTO*, respectively, and average back fat thickness in the Berkshire × Yorkshire F_2 _population. Zhang et al. [19] have reported an association between an SNP in the 5' flanking region of *FTO *and intramuscular fat, but not with average back fat thickness, leaf fat and average daily gain in the Jinhua × Pietrain F_2 _population.

There is no evidence that any of the mutations identified in the pig *FTO *gene cause the observed variation in the traits of interest. However, some of these appear to be in linkage disequilibrium with loci influencing fatness traits. Additional research is required to identify causative mutations in the chromosomal region within and flanking the *FTO *gene.

In addition to association studies involving the *FTO *gene in pig, several genome scans have identified QTL on chromosome 6 in the region harbouring the *RYR1 *gene with effects on fatness traits (see http://www.animalgenome.org/QTLdb/pig.html). However, additional genes linked to *RYR1 *such as *FTO *may be involved in the phenotypic variation of these traits. For example, Mohrmann et al. [32], using F_2 _pigs from crosses between two commercial pig lines, found QTL located close to *RYR1 *for several fatness traits (side fat thickness at the 13/14th rib, external shoulder fat weight, belly weight and loin fat depth) even when adjusting for *RYR1 *genotypes. Paszek et al. [33] detected several fatness QTL (for leaf fat, fat thickness at the 10th rib and average fat thickness) on SSC6, in the interval between markers *SW1841 *- *SW1067 *in F_2 _pigs of Meishan × Yorkshire breeds, and Óvilo et al. [34] have reported a QTL for back fat thickness at the 1st rib in the interval between *SW1057 *and *S0087 *in an Iberian × Landrace intercross. In addition to the *FTO *gene, there are several other candidate genes of interest (*LIPE*, *TGFB1*, *SKI*, *FABP3 *and *ID3*) linked to the *RYR1 *locus [27,35], which may exert effects on the production traits, as shown in Additional file 2 [see Additional file 2].

## Conclusions

SNP FM244720:g.400C>G in pig *FTO *gene was analysed for associations with carcass and growth traits. *FTO *genotypes differ in their effects on several fat deposition traits and some carcass traits, both in Meishan × Pietrain F_2 _family and in commercial pig populations. In the commercial populations, genotypes *CC *and *CG *were associated with a higher average back fat depth, while genotype *GG *was associated with a higher value for some muscling traits. In F_2 _pigs of a Meishan × Pietrain family, heterozygotes with allele *C *from the Pietrain sows and allele *G *from the Meishan boar had significantly higher values for fat-related traits in comparison with homozygotes *G *(from Pietrain) and *G *(from Meishan). The significance of the *FTO *polymorphism for these traits was further supported by the single- and multi-locus models. Results from different association studies between *FTO *polymorphisms and production traits indicate that this gene (and its flanking regions) has a major role in the variation of fatness traits. Further research will be needed to identify candidate/causative mutations in this chromosomal region.

## Competing interests

The authors declare that they have no competing interests.

## Authors' contributions

VD, HB and AS were responsible for the concept, design, data analysis, data interpretation and drafting of the manuscript. PH contributed to molecular genetic analyses and sequencing. RS, JČ and MŠ organized the experiments in the Test Station in Ploskov (Czech Republic) and collected all phenotypic data. AH contributed to genotyping and data analysis. HG organized the experiments in Hohenheim, supervised the genotypic and phenotypic data acquisition and revised the manuscript. All authors have read and approved the final manuscript.

## Supplementary Material

Additional file 1 **Information on PCR and RFLP conditions for SNP analysis in *FTO *and three additional genes (*RYR1*, *LIPE *and *TGFB1*)**. The file shows PCR primers for *FTO*, *RYR1*, *LIPE *and *TGFB1*, amplicon sizes, MgCl_2 _concentrations, annealing temperatures, SNP types and restriction enzymes for RFLP analyses [15,36-39]. Click here for file

Additional file 2 **Associations of the *FTO *polymorphism in combination with linked genes on SSC6 (*RYR1*, *LIPE *and *TGFB1*) with several traits analysed for the Meishan × Pietrain F_2 _pigs (different models used, including single- and multi-locus information)**. Proportions of genetic variance of the four genes (*FTO*, *RYR1*, *LIPE *and *TGFB1*) on selected traits evaluated by single- and multi-locus models are shown. (DOCX 28 kb). Click here for file
