# Supplementary material for: Association between polymorphism in the FTO gene and growth and carcass traits in pig crosses
Source: Genet Sel Evol. 2012 Apr 17;44(1):13. doi: 10.1186/1297-9686-44-13 (PMC3369214; doi:10.1186/1297-9686-44-13)
Supplement: Additional file 1 — Information on PCR and RFLP conditions for SNP analysis in FTO and three additional genes (RYR1, LIPE and TGFB1). The file shows PCR primers for FTO, RYR1, LIPE and TGFB1, amplicon sizes, MgCl2 concentrations, annealing temperatures, SNP types and restriction enzymes for RFLP analyses [15,36-39]. [file 1297-9686-44-13-S1.DOCX]

**Additional file 1- Information on PCR and RFLP conditions for SNP analysis in *FTO* and three additional genes (*RYR1*, *LIPE* and *TGFB1*)**

| Gene: primer pair | Reference  sequence | Primer sequences (5´-3´)^1^ | Frag.  size  (bp) | MgCl_2_  (mM) | Poly-merase | T_a_ (°C) | SNP type | Restrict-ion  enzyme | PCR-RFLP pattern | Reference |
| --- | --- | --- | --- | --- | --- | --- | --- | --- | --- | --- |
| *FTO*: P1 | EU249758 | TCAAGAAGCCTTCCTCGCACTG  TGGGGATCCATGAAGCTCAACA | 435 | 2.0 | LA^2^ | 57 | cf. *FTO*: P2 |  |  | Present study |
| *FTO*: P2 | FM244720  FM244721 | GGCTCTGATGCAAAGTACA  CCATGAAGCTCAACAAAGTTAG | 259 | 2.0 | LA | 57 | 307C>T  400C>G | *Tsc*AI  *Rsa*I | 259/141+118  243+16/216+27+16 | Present study |
| *FTO*: P3 | AM931150 | ACAGGCCCTGAAGAGGAAAG  AGTAACCTGGAGTTCCTGTGG | 397 | 2.0 | *Taq* | 60 | 276T>G | *Tai*I | 397/275+122 | [15] |
| *RYR1*: P1 | X65504 | GTGCTGGATGTCCTGTGTTCCCT  CTGGTGACATAGTTGATGAGGTTTG | 134 | 1.0 | *Taq* | 69 | 192C>T | *Hha*I | 134/84+50 | [36] |
| *RYR1*: P2 | M91456 | TCCAGTTTGCCACAGGTCCTACCA  ATTCACCGGAGTGGAGTCTCTGAG | 660 | 1.0 | *Taq* | 53 | 990C>T | *Hgi*AI | 520+140/357+163+  140 | [37] |
| *LIPE*: P1 | AJ224692 | CGCACRATGACACAGTCGCTGGT  CAGGCAGCGRCCRTAGAAGCA | 498 | 2.0 | *Taq* | 60 | 433A>G | *Hsp*92I | 308+190/  241+190+67 | [38] |
| *TGFB1*: P1 | AJ621785 | GTGCGGCAGCTCTACATTGACT  TTGCGGCCCACGTAGTACAC | 1156 | 1.5 | LA | 55 | 180A>G | *Ava*II | 735+241+104+46+30/  586+241+149+104+  46+30 | [39] |

^1^R stands for A and G

^2^LA DNA polymerases mix (Top-Bio, Prague, Czech Republic)
