# Supplementary material for: Association between polymorphism in the FTO gene and growth and carcass traits in pig crosses
Source: Genet Sel Evol. 2012 Apr 17;44(1):13. doi: 10.1186/1297-9686-44-13 (PMC3369214; doi:10.1186/1297-9686-44-13)
Supplement: Additional file 2 — Associations of the FTO polymorphism in combination with linked genes on SSC6 (RYR1, LIPE and TGFB1) with several traits analysed for the Meishan × Pietrain F2 pigs (different models used, including single- and multi-locus information). Proportions of genetic variance of the four genes (FTO, RYR1, LIPE and TGFB1) on selected traits evaluated by single- and multi-locus models are shown. (DOCX 28 kb). [file 1297-9686-44-13-S2.DOCX]

**Additional file 2 - Associations of the *FTO* polymorphism in combination with linked genes on SSC6 (*RYR1*, *LIPE* and *TGFB1*) with several traits analysed for the Meishan x Pietrain F_2_ pigs (different models used, including single- and multi-locus genotype information)**

| Trait | Model version | *FTO* | | | | *RYR1* | | *LIPE* | | | *TGFB1* | | | Initial model^3^ | | | Complete model | |
| --- | --- | --- | --- | --- | --- | --- | --- | --- | --- | --- | --- | --- | --- | --- | --- | --- | --- | --- |
|  |  | 74.6^2^ | | | | 96.7 (+22.1) | | 98.6 (+24.0) | | | 99.5 (+24.9) | | |  |  |  |  |  |
|  | s/m^1^ | *F* ratio | *P* ^4^ | | *F* ratio | | *P* | | *F* ratio | *P* | | *F* ratio | *P* | *n* | *mean* | *r^2^*(%)^5^ | *r^2^*(%) | VR (%)^6^ |
| Fat cuts (%) | s | 5.98 | .003** | | --^7^ | | -- | | -- | -- | | -- | -- | 307 | 18.4 | 25.3 | 28.2 | 3.24 |
|  | s | -- | -- | | **24.51** | | <.**001***** | | -- | -- | | -- | -- |  |  |  | 35.9 | 13.63 |
|  | s | -- | -- | | -- | | -- | | -- | -- | | 22.84 | <.001*** |  |  |  | 35.3 | 12.79 |
|  | m | 2.59 | ns | | **4.87** | | **.008**** | | 2.55 | ns | | 4.15 | .017* |  |  |  | 39.1 | 16.26 |
| Back fat depth on  *m.l.l.t.* at 13^th^/14^th^  rib (mm) | **s** | **4.76** | **.009**** | | -- | | -- | | -- | -- | | -- | -- | 307 | 22.0 | 27.6 | 29.9 | 2.46 |
|  | s | -- | -- | | 11.62 | | <.001*** | | -- | -- | | -- | -- |  |  |  | 32.9 | 6.65 |
|  | s | -- | -- | | -- | | -- | | -- | -- | | 9.83 | <.001*** |  |  |  | 32.2 | 5.59 |
|  | m | **5.10** | **.007**** | | 3.06 | | .049* | | 2.94 | ns | | 1.81 | ns |  |  |  | 36.5 | 9.81 |
| Lean cuts (%) | s | 8.08 | <.001*** | | -- | | -- | | -- | -- | | -- | -- | 307 | 45.4 | 19.5 | 23.7 | 4.53 |
|  | s | -- | -- | | **27.55** | | <.**001***** | | -- | -- | | -- | -- |  |  |  | 32.1 | 15.12 |
|  | s | -- | -- | | -- | | -- | | -- | -- | | 27.13 | <.001*** |  |  |  | 32.0 | 14.91 |
|  | m | 2.10 | ns | | **4.05** | | **.018*** | | 2.71 | ns | | 3.80 | .023* |  |  |  | 35.4 | 17.52 |
| Weight of ham  relative to half  carcass weight (%) | s | 10.32 | <.001*** | | -- | | -- | | -- | -- | | -- | -- | 307 | 18.8 | 19.2 | 24.4 | 5.88 |
|  | s | -- | -- | | 25.34 | | <.001*** | | -- | -- | | -- | -- |  |  |  | 31.0 | 14.04 |
|  | s | -- | -- | | -- | | -- | | -- | -- | | **31.74** | <.**001***** |  |  |  | 33.4 | 17.10 |
|  | m | 2.05 | ns | | 1.58 | | ns | | 4.14 | .017* | | **7.53** | <.**001***** |  |  |  | 36.4 | 19.16 |
| Meat area on *m.l.l.t.*  at 13^th^/14^th^ rib (cm^2^) | **s** | **7.43** | **<.001***** | | -- | | -- | | -- | -- | | -- | -- | 307 | 29.3 | 13.9 | 18.0 | 4.14 |
|  | s | -- | -- | | 5.30 | | .005** | | -- | -- | | -- | -- |  |  |  | 16.9 | 2.80 |
|  | s | -- | -- | | -- | | -- | | -- | -- | | 4.88 | .008** |  |  |  | 16.7 | 2.54 |
| pH *m.l.l.t.* 45 min  pm | s | 17.16 | <.001*** | | -- | | -- | | -- | -- | | -- | -- | 307 | 5.68 | 3.5 | 13.5 | 9.78 |
|  | s | -- | -- | | **86.97** | | <.**001***** | | -- | --- | | -- | -- |  |  |  | 39.2 | 36.59 |
|  | s | -- | -- | | -- | | -- | | 32.34 | <.001*** | | -- | -- |  |  |  | 20.8 | 17.38 |
|  | s | -- | -- | | -- | | -- | | -- | -- | | 71.29 | <.001*** |  |  |  | 34.9 | 32.06 |
| Conductivity *m.l.l.t.*  24 h pm | s | 19.85 | <.001*** | | -- | | -- | | -- | -- | | -- | -- | 307 | 7.37 | 4.5 | 15.8 | 11.23 |
|  | s | -- | -- | | **89.59** | | <**.001***** | | -- | -- | | -- | -- |  |  |  | 40.5 | 37.29 |
|  | s | -- | -- | | -- | | -- | | 35.83 | <.001*** | | -- | -- |  |  |  | 23.1 | 18.95 |
|  | s | -- | -- | | -- | | -- | | -- | -- | | 79.45 | <.001*** |  |  |  | 37.9 | 33.62 |
|  | m | -- | -- | | **12.28** | | <**.001***** | | -- | -- | | 5.48 | .005** |  |  |  | 42.7 | 39.13 |
| G6P^8^  (units/g tissue) | s | **5.78** | **.003**** | | -- | | -- | | -- | -- | | -- | -- | 306 | .237 | 15.0 | 18.2 | 3.12 |
|  | m | **8.45** | <.**001***** | | 3.48 | | .032* | | -- | -- | | -- | -- |  |  |  | 20.1 | 4.73 |
|  | m | **9.11** | <.**001***** | | -- | | -- | | -- | -- | | 4.03 | .019* |  |  |  | 20.4 | 5.07 |
| Average area per red  muscle fibre (µm^2^) | s | 8.54 | <.001*** | | -- | | -- | | -- | -- | | -- | -- | 300 | 4740 | 10.8 | 15.8 | 4.93 |
|  | s | -- | -- | | **37.69** | | <.**001***** | | -- | -- | | -- | -- |  |  |  | 29.3 | 20.14 |
|  | **s** | **--** | | **--** | **--** | | **--** | | **--** | **--** | | 27.27 | <.001*** |  |  |  | 25.0 | 15.29 |

**Footnotes to Additional file 2**

Only the traits associated with *FTO* on *P* < 0.01 significance level in the single-locus model were included. Traits associated on similar level were excluded in case of high correlation with any of the chosen traits to reduce the table size

^1^ s = single-locus model; m = multi-locus model, calculated in case of significantly increased genetic variance proportion using covariance

^2^ Position of locus within the linkage map in cM (value within brackets represents the distance to *FTO*)

^3^ Initial model including fixed effects sex, season (two-month classes), litter number and slaughter age as continuous linear effect

^4^ *P* = *F* test probability; * *P* < 0.05; ** *P* < 0.01; *** *P* < 0.001

^5^ *r^2^* (%) = determination coefficient

^6^ VR (%) = variance reduction (%) with the inclusion of the genetic variable/s in the initial model

^7^ -- = locus not included in the model

^8^ G6P = glucose-6-phosphate dehydrogenase from subcutaneous inner backfat layer

Numbers in bold characters indicate the polymorphisms that contributed most to genetic variance using a single- or a multi-locus model

*m.l.l.t.* – *musculus longissimus lumborum et thoracis*
